# Accuracy of serum luteinizing hormone and serum testosterone measurements to assess the efficacy of medical castration in prostate cancer patients

**DOI:** 10.1186/s12929-017-0386-0

**Published:** 2017-10-22

**Authors:** Juan Morote, Imma Comas, Roser Ferrer, Jacques Planas, Anna Celma, Lucas Regis

**Affiliations:** 1 Department of Urology, Vall d’Hebron Hospital, Universidad Autonoma de Barcelona, 14 Po Vall d’Hebron 119-129, 00173 Barcelona, Spain; 2 Department of Biochemistry, Vall d’Hebron Hospital, Universitat Autònoma de Barcelona, Barcelona, Spain

**Keywords:** Prostate cancer, Androgen deprivation therapy, LH-RH agonist, Testosterone, Luteinizing hormone

## Abstract

**Background:**

Luteinizing hormone-releasing hormone (LH-RH) agonists are the standard for androgen deprivation therapy (ADT) in prostate cancer (PCa) patients. Current guidelines recommend serum testosterone measurement to assess the efficacy of ADT and to define castration resistance. However, serum testosterone does not reflect the exclusive effect of castration due to its extratesticular production. The aim of this study is to analyze if serum LH reflects better than serum testosterone the activity of LH-RH agonists.

**Methods:**

Serum LH and serum testosterone were measured with chemiluminescent immunoassay (CLIA) in a cohort study of 1091 participants: 488 PCa patients “on LH-RH agonists”, 303 “off LH-RH agonist” in whom LH-RH agonists were withdrawn, and 350 men with PCa suspicion “no LH-RH agonist” who never received LH-RH agonists. In a validation cohort of 147 PCa patients, 124 on “LH-RH agonists” and 19 “off LH-RH agonists”, serum testosterone was also measured with liquid chromatography and tandem mass spectrometry (LC MSMS).

**Results:**

The area under the curve (AUC) to distinguish patients “on versus off LH-RH agonists” was 0.997 for serum LH and 0.740 for serum testosterone, *P* < 0.001. The 97.5 percentile of serum LH in patients “on LH-RH agonists” was 0.97 U/L, been the most efficient threshold 1.1 U/L. The AUCs for serum LH, testosterone measured with CLIA and with LC MSMS, in the validation cohort, were respectively 1.000, 0.646 and 0.814, *P* < 0.001. The efficacy to distinguish patients “on versus off LH-RH agonists” was 98.6%, 78.3%, and 89.5% respectively, using 1.1 U/L as threshold for serum LH and 50 ng/dL for serum testosterone regardless the method.

**Conclusions:**

Serum LH is more accurate than serum testosterone regardless the method, to distinguish patients “on versus off LH-RH agonists”. The castrate level of serum LH is 1.1 U/l. These findings suggest that assessment of LH-RH agonist efficacy and castration resistance definition should be reviewed.

## Background

Medical castration with LH-RH agonists is currently the standard of ADT in patients with PCa [1]. PCa guidelines recommend measurements of testosterone in serum or plasma to assess the efficacy of ADT and also to define the castration resistance when biochemical or clinical progression appears [2]. The current castration level of serum testosterone is up to 50 ng/dl; however, microelevations over this level are observed in up to 25% of PCa patients on LH-RH agonist [3]. The origin of these microelevations of serum testosterone remains unclear. They may be due to an inadequate castration or a response to a new administration of LH-RH agonist. An extratesticular over-production of testosterone in the adrenal cortex or in the tumour cells may be also considered [4]. These microelevations of serum testosterone have been associated with poorer disease outcomes [5, 6].

The behaviour of the hypothalamic-pituitary-gonadal axis varies according to the type of castration. After surgical castration elevated levels of serum LH are present while medical castration promptly induces a severe reduction in serum LH due the complete blockade of pituitary gland receptors [7]. After an acute LH-RH agonist administration an initial over-production of serum LH precedes the maximal blockade of pituitary LH receptors. This secondary flare of serum testosterone may induce clinical consequences avoidable with prophylactic administration of anti-androgens [8]. In contrast, LH-RH antagonist administration rapidly reduces serum LH and testosterone as surgical castration does [1]. The measurement of serum LH is sensible to assess the effect of LH-RH agonists or antagonists [9].

The method to measure serum testosterone is crucial [10]. Nowadays chemiluminescent immunoassays (CLIAs) are worldwide used because they are sensitive, automatable, fast and cheap. However, due to their lack of accuracy and reproducibility, they are not recommended especially to measure low levels of testosterone [11]. The only recommended methods to measure serum testosterone are those based on previous chromatography and mass spectrometry [12].

Our hypothesis is that serum LH measurements are more accurate than serum testosterone to assess the activity of LH-RH agonists. The main objective of this study was to demonstrate that serum LH correlates better than serum testosterone with LH-RH agonist’s activity. Secondary objectives were to define the castrate level of serum LH and to validate previous results when serum testosterone was measured with liquid chromatography and tandem mass spectrometry (LC MSMS).

## Methods

### Design and participants

We prospectively measured serum LH and serum testosterone in 1238 men divided into a study cohort of 1091 participants and a validation cohort of 147. In the study cohort, 791 participants had histologically confirmed PCa and received LH-RH agonist. A subset of 488 patients “on LH-RH agonist” were on continuous treatment for longer than 3 month, and 303 patients “off LH-RH agonists” received LH-RH agonists for 24 to 36 months as a neo-adjuvant treatment to radiotherapy. In all of these participants the activity of LH-RH agonist finished at least 1 month before the biochemical measurements. A control group of 350 men “no LH-RH agonist” with similar age was randomly selected from 864 men scheduled to prostatic biopsy due to PCa suspicion. Men of this control group never received LH-RH agonists or 5-alpha reductase inhibitors. The validation cohort, where serum testosterone was measured with CLIA and LC MSMS, included 124 PCa patients “on LH-RH agonists” and 19 “off LH-RH agonists”.

### Measurements of serum LH and testosterone

Blood collection took place between 8:00 and 10:00 am, and serum was extracted from the samples. LH was measured with CLIA using the automated platform Advia-Centaur XPi® (Siemens Inc., NY). The lowest level of quantification (LLOQ) was 0.12 U/L, and according to the manufacturer the intra-assay coefficient of variation ranged between 2,3% and 3.0%, and the inter-assay coefficient of variation between 1,5% and 2.9%. Testosterone was also measured with CLIA, using the automated platform Advia-Centaur XPi® (Siemens Inc., NY). The LLOQ was 10 ng/dL, and according to the manufacturer, the intra-assay coefficient of variation ranged between 2.3% and 6.2%, and the inter-assay coefficient of variation between 2.7% and 6.9% (Manufacturer information: 10629910_ES Rev. U, 2014–08, 1–18). Testosterone was also measured with LC MSMS in the validation cohort. An ultra high-pressure liquid chromatography with the 1290 Infinity Binary LC System (Agilent Technologies, Santa, CA) was performed. The system is connected in parallel to a tandem mass spectrometry using the 6430 Series Tripe Quadruple LC/MS System (Agilent Technologies, Santa Clara, CA). The LLOQ was 2 ng/dL and thee intra-assay coefficient of variation ranged from 4% to 5% and the inter-assay coefficient of variation between 7% and 8%, at levels of ST below 50 ng/dL [13].

### Statistic analysis

The behaviours of serum LH and serum testosterone were analysed in every group using the Shapiro test. The 2.5, 25, 50, 75, and 97.5 percentiles of serum LH and testosterone were estimated and also after their logarithmic transformation if no kurtotic distribution was observed [14]. Comparisons between two or more groups were carried out with the Mann Witney U test and the Kruscal-Wallis test. ROC curves were generated and areas under the curve (AUC) were calculated and compared. The castration level of serum LH was estimated from the study cohort, and sensitivity, specificity and positive and negative predictive values (PPV and NPV) were calculated. Up to 50 ng/dl was considered the castration level of serum testosterone regardless of the method used [2]. This analysis was carried out using the Statistical Package for the Social Sciences (SPSS), version 20.

## Results

Serum LH and serum testosterone did not follow a kurtotic distribution in any group, *P* < 0.001. The median age was 70 years in patients “on LH-RH agonists”, 71 years in patients “off LH-RH agonists” and 70 years in men “no LH-RH agonists”, *P* = 0.367. Statistics analysing serum LH and serum testosterone levels in the study cohort are summarized in Table 1. The median serum LH was below the LLOQ in patients “on LH-RH agonist”. In fact, the 75th percentile was below 0.12 U/L. In contrast, the median of serum LH in the group of patients “off LH-RH agonists” was higher than the observed in men who never received an LH-RH agonist, *P* < 0.001. The trend for serum testosterone was somewhat different. The lowest level of testosterone was observed in patients “on LH-RH agonists”. Patients “off LH-RH agonists” had a moderately high level of serum testosterone, *P* < 0.001, while a much higher amount was recorded in men who never received LH-RH agonist, *P* < 0.001.

**Table 1:** Levels of serum LH and testosterone according to LH-RH agonist activity

Group	Serum LH^a^				Serum testosterone			
	p 50	p 25-p75	p 2.5	p 97.5	p 50	p 25-p 75	p 2.5	p 97.5
On LH-RH agonist	0.12	0.12–0.12	0.12	0.85	32.7	23.8–44.9	11.6	78.4
Off LH-RH agonist	8.69	5.18–14.79	1.17	37.42	69.9	32.4–131.7	10.0	542.6
Never LH-RH agonist	4.45	3.11–4.46	1.39	16.51	385.6	294.5–506.4	182.9	745.7
*P* value On vs Off	0.0001				0.0001			
*P* value On vs No	0.0001				0.0001			
*P* value Off vs No	0.0001				0.0001			

^a^Luteinizng hormone; Values of LH and testosterone expressed as percentiles

**Fig. 1:**
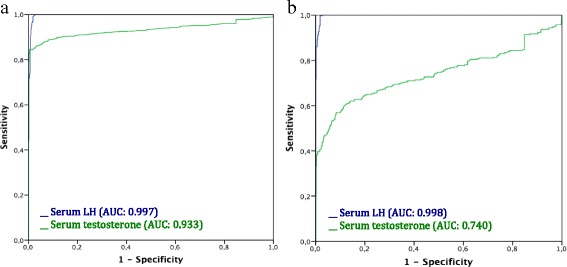
ROC curve analysis of serum LH and serum testosterone according to the LH-RH agonist activity; (**a**) including patients off and no LH-RH agonist, and (**b**) including only patients off LH-RH agonist

The ROC curves of serum LH and serum testosterone to distinguish patients “on LH-RH agonist” from those “off or no LH-RH agonists” are presented in Fig. 1. The AUCs of serum LH were 0.998 and 0.997 respectively, while those of serum testosterone were 0.993 and 0.740. The efficacy of serum LH to distinguish patients “on LH-RH agonists” from those “off or no LH-RH agonists” was significantly higher than the observed for serum testosterone, *P* < 0.001.

The 97.5th percentile of serum LH in the subset of patients “on an LH-RH agonist” was 0.85 U/L and increased to 0.97 U/L after its logarithmic transformation. However, the most efficient threshold of serum LH to distinguish patients “on LH-RH agonists” from those “off or no LH-RH agonists” was 1.1 U/L. The efficacy parameters of 1.1 U/L of serum LH and 50 ng/dL of serum testosterone are presented in Table 2. The castrate level of serum LH distinguished 99.1% of patients while serum testosterone 75.7%. The probability to be “on LH-RH agonist” when serum LH was lower than 1.1 U/L was 99.8%, while the likelihood of being “off or no LH-RH agonists” when serum LH was higher than 1.1 U/L was 98.1%. The PPV and NPV of serum testosterone were 78.5% and 75.1%, respectively.

**Table 2:** Serum LH and testosterone efficacy parameters according to “on versus off LH-RH agonist” in the cohort study

Marker	Sensitivity	Specificity	Efficiency	PPV^a^	NPV^b^
Serum LH^c^	482/488 (98.8)	302/303 (99.7)	784/791 (99.1)	482/483 (99.8)	302/308 (98.1)
Serum testosterone	408/488 (83.6)	191/303 (63.0)	599/791 (75.7)	408/520 (78.5)	191/271 (75.7)

^a^ *PPV* Positive predictive value, ^b^ *NPV* Negative predictive value, ^c^ *LH* Luteinizing hormone

**Fig. 2:**
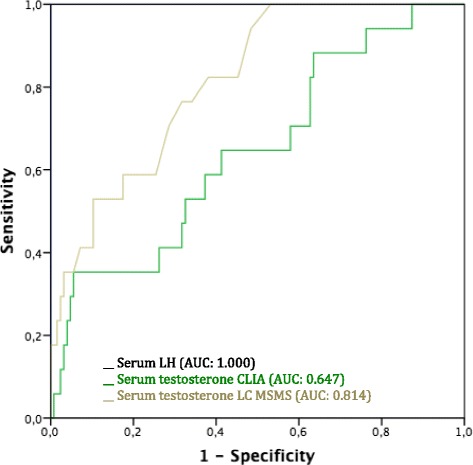
ROC curve analysis of serum LH and serum testosterone, measured with CLIA or LC MSMS, according to the LH-RH agonist activity

The ROC curves of serum LH and serum testosterone in the validation cohort are presented in Fig. 2. The AUC of serum LH was 1.000, been 0.647 when serum testosterone was measured with CLIA and 0.814 when it was measured with LC MSMS, *P* < 0.001.

The parameters of efficacy for 1.1 U/L of serum LH and 50 ng/dL for serum testosterone measured with CLIA and LC MSMS are presented in Table 3. The efficacy rates for distinguishing patients “on versus off LH-RH agonist” were 98.6%, 78.3% and 89.5%, respectively. Serum LH PPV and NPV were 100% and 89.5% respectively. Serum testosterone PPV and NPV were 90.6% and 23.1% when it was measured with CLIA and 91.1% and 62.5% respectively when it was measured with LC MSMS.

**Table 3:** Serum LH and testosterone efficacy parameters according to “on versus off LH-RH agonist” in the validation cohort

Marker	Sensitivity	Specificity	Efficiency	PPV^a^	NPV^b^
Serum LH^c^	124/126 (98.4)	17/17 (100)	141/142(98.6)	124/124 (100)	17/17 (89.5)
Serum testosterone (CLIA)^d^	106/126 (84.1)	6/17 (35.3)	112/143 (78.3)	106/117 (90.6)	6/26 (23.1)
Serum testosterone (LC MSMS)^e^	123/126 (97.6)	5/17 (29.4)	128/143 (89.5)	123/135 (91.1)	5/8 (62.5)

^a^ *PPV* positive predictive value, ^b^ *NPV* negative predictive value, ^c^ *LH* Luteinizing hormone, ^d^ *CLIA* Chemiluminiscent immunoassay, ^e^ *LC MSMS* Liquid chromatography tandem mass espectrometry

## Discussion

This is the first study to analyse the accuracy of serum LH and serum testosterone as markers of medical castration in patients undergoing LH-RH agonists. Our results suggest that serum LH can better distinguish patients on LH-RH agonist from those off LH-RH agonist than serum testosterone does distinguish.

Under normal conditions, between one and 2% of serum testosterone amount is synthesised in the adrenal glands [15]. Evidence exists that testosterone may also be produced by prostate cancer cells as well [4, 16]. Microelevations of serum testosterone in patients undergoing LH-RH agonists are therefore not always secondary to an inadequate castration [3].

The acute injection of an LH-RH agonist prompts the marked release of LH, whereas prolonged administration produces inhibitory effects. Repeated injections of LH-RH agonists and the use of depot preparations suppress the function of pituitary gonadotrophs and the secretion of gonadotropins [17]. As a consequence of this serum LH behaviour following LH-RH agonist administration, serum testosterone level increases due to a previous increase of serum LH; decreasing to castration levels usually occurs within 4 weeks after treatment [18]. The initial rise in serum testosterone does not take place if LH-RH antagonists are administered and castration levels of serum testosterone are reached within a few hours, which is similar to what happens after surgical castration [19].

The behaviour of serum testosterone and serum LH after LH-RH agonist withdrawal has also been studied. The overall impression is that serum testosterone recovery may be slow, especially following a long administration of LH-RH agonists, while serum LH quickly increases after LH-RH withdrawal, reaching levels greater than those observed before LH-RH agonist administration [18, 20, 21]. Serum LH measurement has been used to analyse the switch from LHRH antagonists to LH-RH agonists; it has been noted that it remains low and stable during this manoeuvre [22, 23].

The first objective of our study was to demonstrate that serum LH correlates better than serum testosterone with the active treatment of LH-RH agonists. The ROC curves clearly indicate that the ability of serum LH to distinguish between patients currently on LH-RH agonists and those who are off LH-RH agonists is superior to that of serum testosterone, even when it is measured using LC MSMS. There is no information in the literature to compare our results with. In our study we confirm, though, a similar behaviour of serum testosterone when measured using CLIA as we had observed in our previous studies [3, 24].

Our second objective was to define the castration level of serum LH in patients undergoing LH-RH agonist, which had never been described before. We estimated this level from the 97.5 percentile of serum LH distribution in patients on LH-RH agonist, after its logarithmic transformation. The overlap of serum LH values in patients “on LH-RH” agonists and “off LH-RH” agonist was also considered. We finally established in 1.1 U/l the most efficient threshold, which was able to classify adequately the 99.1% of patients in the study cohort and the 98.6% in the validation cohort.

Finally, our tertiary objective was to validate the previous results in a validation cohort of patients in whom serum testosterone was measured simultaneously with CLIA and LC MSMS as reference method [11]. Here, the ROC curves reproduced similar results than those observed in the study cohort. The effectiveness of serum LH to distinguish patients “on versus off” LH-RH agonists was 98.6%, which was significantly higher than the 78.3% and 89.5% observed when testosterone was measured respectively with CLIA and LC MSMS.

Our data indicated that serum LH is better marker of medical castration than serum testosterone regardless the method. A limitation of our study was the difficulty to know the exact moment when a patient loss the effect of LH-RH agonist, especially in those patients using long-depot formulations. The behaviour of serum testosterone after LH-RH agonist withdrawal is well known trough the intermittent ADT studies [25]; however, less is known after long periods of continuous LH-RH agonist treatment [21]. Moreover, there is a lack of knowledge about the exact behaviour of serum LH after LH-RH agonist withdrawal because the actual CLIAs were not always used in the past [26]. The dynamics of serum LH after LH-RH agonist withdrawal remains unclear and should be analysed in well-designed prospective longitudinal studies.

## Conclusions

Serum LH is better marker of medical castration than serum testosterone is, regardless the method of measurement. Serum LH is able to distinguish patients “on versus off” LH-RH agonists with higher accuracy than serum testosterone does. We propose 1.1 U/l as the castrate level of serum LH, which is able to distinguish patients “on versus off” LH-RH agonist activity with an efficacy similar to 99%. Based on these findings, the assessment of LH-RH agonists efficacy and especially the definition of castration resistance should be redefined.

## Abbreviations

ADT: Androgen deprivation therapy
AUC: Area under the curve
CLIA: Chemiluminiscent immunoassay
LC MSMS: Liquid chromatography tandem mass spectrometry
LH: Luteinizing hormone
LH-RH: Luteinizing hormone releasing hormone
LLOQ: Lowest level of quantification
NPV: Negative predictive value
PCa: Prostate cancer
PPV: Positive predictive value
ROC: Receiver operating characteristic
